# High-flow nasal cannula can’t be considered non-inferior to noninvasive ventilation in patients with chronic obstructive pulmonary disease who develop respiratory failure after extubation

**DOI:** 10.1186/s13054-020-03363-x

**Published:** 2020-11-23

**Authors:** Robert V. Curtis, Badih A. Kabchi, Shehabaldin Alqalyoobi

**Affiliations:** Present Address: Division of Pulmonary, Critical Care and Sleep Medicine, Department of Internal Medicine, East Carolina University-Brody School of Medicine, Mail Stop 628, 3E-149, Greenville, NC 27834 USA

We read the article published recently in Critical Care by  Tan et al. with great interest. We appreciate their effort to evaluate high flow nasal cannula (HFNC) usage in post-extubated chronic obstructive pulmonary disease (COPD) patients with respiratory failure [1]. A non-inferiority study is a reasonable approach given NIV has shown benefit in post-extubation studies [2–4]. Unlike superiority trials, non-inferiority studies establish non-inferiority by rejecting a null hypothesis that the tested treatment is worse than the comparator by a pre-established minimum difference (non-inferiority cutoff or delta) based on results from prior studies [5]. However, several issues in this study prevent us from reaching this conclusion.

First, Tan et al. anticipated an NIV failure rate of 22% based on prior trials. The authors determined a delta of 9% for non-inferiority cutoff. They found that the experimental group (HFNC) had failure rates less than the control group (NIV), 22.7% vs. 28.6%, respectively. The absolute risk difference was − 5.8% (CI 95%; − 23.8 to 12.5). Since the CI range extends beyond the predetermined non-inferiority cut-off, inferiority is still a possibility and the study should be considered inconclusive [5]. For further clarification, we created a forest plot to visualize the primary outcomes CIs in relation to the delta point (Fig. 1).

**Fig. 1:**
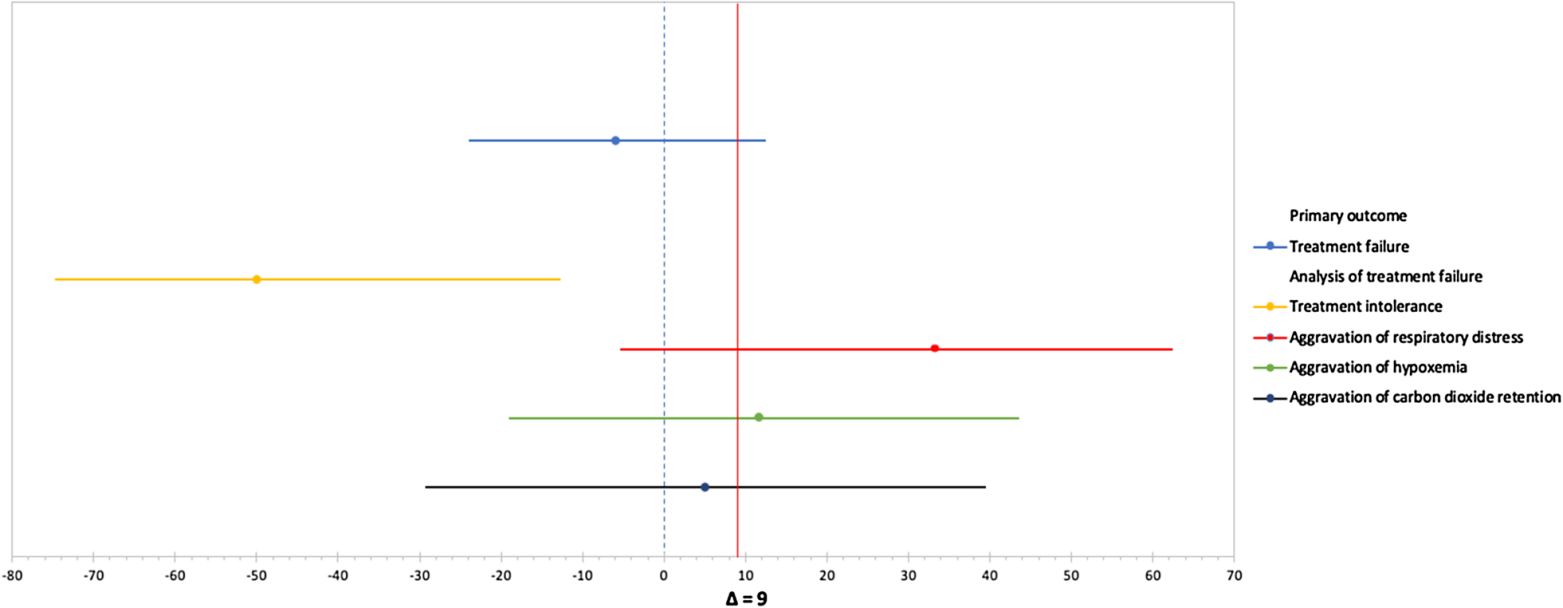
Forest plot of confidence intervals (CI) for primary outcome and the causes of treatment failure

Second, the suggested sample size of 44 subjects per group seems insufficient. We don’t have a description of the calculations used by Tan et al., but using their assumptions we calculated that at least 216 patients per arm would be required to prove non-inferiority with a difference of less than 9%, an alpha of 0.05, and a beta of 0.2 (it is worth noting that in the paper it is stated an alpha of 0.5 instead of 0.05, which we think is a typo since such a large error margin is not considered acceptable). A trial with a similar design referenced by Tan et al. [4] calculated a sample size of 300 patients per arm using similar assumptions.

Third, the failure rate in the NIV arm is higher than expected (28.6% vs 22%, OR = 1.3), and is higher than other previous studies [2–4]. This may create bias in favor of non-inferiority and should have been discussed further in the paper.

In summary, we conclude that the results of Tan et al.’s study can’t prove non-inferiority of HFNC compared to NIV, although it doesn’t exclude it either. Besides, further clarification regarding the sample size is required.

## Data Availability

Not applicable.
