# Supplementary material for: High-flow nasal cannula can’t be considered non-inferior to noninvasive ventilation in patients with chronic obstructive pulmonary disease who develop respiratory failure after extubation
Source: Crit Care. 2020 Nov 23;24:659. doi: 10.1186/s13054-020-03363-x (PMC7684711; doi:10.1186/s13054-020-03363-x)
Supplement: Related texts

# Reply: Authors’ response

**Authors:** Jiayan Sun, Dingyu Tan, Joseph Harold Walline, Jun Xu

Dear Editor,

We would like to thank Drs. Curtis, Kabchi and Alqalyoobi for their detailed comments and helpful feedback. We should first emphasize that the primary endpoint in our study was a composite of re-intubation and switching between non-invasive ventilation (NIV) and high-flow nasal cannula (HFNC), which would better reflect a real-life treatment failure of HFNC or NIV [6]. This composite endpoint had a significant impact on setting the estimated NIV failure rate and sample size. Most previous studies have defined a NIV failure as only tracheal intubation. In our study, the NIV failure rate was 28.6%, but, again, this was a composite of the re-intubation and treatment switch rates, leading to a higher overall NIV failure rate in our study.

In the study with a sample size of 300 mentioned in the letter, the baseline re-intubation rate for the two groups was set at the same value, though the non-inferiority cutoff value was similar to our study [4]. However, recent studies have shown that the treatment failure rate of HFNC is 4–12% lower than that of NIV [4, 7, 8], which significantly reduced the sample size required for our study. With reference to the trial of Kullberg et al. [9], according to different baseline failure rates (maximum 12% difference), we calculated that at least 88 patients would be required to assess a non-inferiority cutoff at 9% using an *α* = 0.05 (0.50 alpha was indeed a typo) and a *β* = 0.20.

Combining the statistical trend of high probability, we were careful in our paper to conclude that HFNC after extubation did not result in increased rates of treatment failure compared with NIV, though the CI range extends beyond the predetermined non-inferiority cut-off. Nevertheless, we believe the data we presented is a helpful first step to comparing HFNC with NIV in chronic obstructive pulmonary disease (COPD) patients. As the first randomized controlled trial to compare the failure rate of HFNC to NIV in patients with COPD after invasive ventilation, there may be different interpretations of the ideal sample size and test values. We set the feasible minimum sample size according to our calculations and found that the absolute risk difference between HFNC and NIV was − 5.8% (CI 95%; − 23.8 to 12.5). We agree that future studies with larger sample sizes should be able to narrow the CI range and help determine the best use of HFNC.
